# Estradiol and Tamoxifen Induce Cell Migration through GPR30 and Activation of Focal Adhesion Kinase (FAK) in Endometrial Cancers with Low or without Nuclear Estrogen Receptor α (ERα)

**DOI:** 10.1371/journal.pone.0072999

**Published:** 2013-09-09

**Authors:** Chia-Lung Tsai, Hsien-Ming Wu, Chiao-Yun Lin, Yi-Jun Lin, Angel Chao, Tzu-Hao Wang, Swei Hsueh, Chyong-Huey Lai, Hsin-Shih Wang

**Affiliations:** 1 Department of Obstetrics and Gynecology, Chang Gung Memorial Hospital, Lin-Kou Medical Center, Chang Gung University, Taoyuan, Taiwan; 2 Genomic Medicine Research Core Laboratory, Chang Gung Memorial Hospital, Taoyuan, Taiwan; 3 Department of Pathology, Chang Gung Memorial Hospital, Lin-Kou Medical Center, Chang Gung University, Taoyuan, Taiwan; 4 Graduate Institute of Clinical Medical Sciences, College of Medicine, Chang Gung University, Taoyuan, Taiwan; II Università di Napoli, Italy

## Abstract

Estrogens and tamoxifen (an antiestrogen) exert their actions by activation of estrogen receptor (ER) through genomic and non-genomic mechanisms and are implicated in the development of endometrial cancer. Previous reports have demonstrated that estradiol and tamoxifen induce proliferation of human endometrial cancer cells through GPR30 (non-genomic ER) signaling pathway. Herein, we demonstrate that phosphorylation of focal adhesion kinase (FAK) is involved in cell migration induced by estradiol, tamoxifen and G1 (a GPR30 agonist) through the transmembrane ER (GPR30) in endometrial cancer cell lines with or without ERα (Ishikawa and RL95-2). Additionally, the GPR30-mediated cell migration was further abolished by administration of either specific RNA interference targeting GPR30 or an FAK inhibitor. Moreover, we have validated that the signaling between GPR30 and phosphorylated FAK is indeed mediated by the EGFR/PI3K/ERK pathway. Clinically, a significant correlation between levels of GPR30 and phophorylated FAK (pFAK) observed in human endometrial cancer tissues with low or without ERα further suggested that estrogen-induced phosphorylation of FAK and cell migration were most likely triggered by GPR30 activation. These results provided new insights for understanding the pathophysiological functions of GPR30 in human endometrial cancers.

## Introduction

Estrogens bind and activate estrogen receptors (ER) to regulate the transcription of target genes [1] via genomic and non-genomic mechanisms. The genomic (or classic) estrogen-evoked responses are through nuclear receptors ERα and ERβ [2]. ERα is the receptor responsible for 17β-estradiol (E2)-induced signaling, whereas function of ERβ is opposed to that of ERα [3]. The genomic ER signaling functions as ligand-dependent transcription factors that directly bind to estrogen response elements (EREs) in the promoter region of target genes and usually takes hours to days to produce physiological effects in cells [4]. In contrast, non-genomic ER signaling is through a transmembrane receptor for estrogen [2], [5].

The G protein-coupled receptor 30 (GPR30) is a functional membrane receptor involved in non-genomic estrogen signaling [6]–[8]. The GPR30-mediated estrogen signaling stimulates cAMP production and intracellular Ca^2+^ mobilization, and subsequently activates various kinases that contributes to cell growth and migration [2], [9], [10]. In breast cancer cells lacking ERs, GPR30 mediates up-regulation of the c-fos protein in the presence of estrogens, leading to promotion of cell proliferation [11]. Additionally, both estradiol and tamoxifen induce the expression of *c-fos* and cell proliferation through GPR30 (non-genomic ER) signaling pathway in various cancers [12], [13]. Moreover, it is also evident that overexpression of GPR30 indicates poor prognosis of endometrial, ovarian and breast cancers [14]–[16].

Cell migration is required for invasion of tumors. Focal adhesion kinase (FAK), a non-receptor tyrosine kinase controlling cellular signaling pathways of cell migration [17], is involved in the formation and turnover of focal adhesion sites [18], [19]. In addition, overexpression of FAK has been demonstrated to indicate invasive potential and poor prognosis in various human cancers [20].

Prolonged exposure to endogenous or exogenous estrogens and tamoxifen (an estrogen antagonist) is one of the risk factors for cell proliferation and migration in endometrial cancer [21]–[23]. However, the effects of estrogen on cell migration of endometrial cancers with low or without ERα were not previously explored, though previous studies have shown that estrogen induces a rapid phosphorylation of FAK in endometrial stroma and cancer cells [23]. Noteworthily, it is still unclear whether estrogens and tamoxifen simply stimulate the proliferation of endometrial cells *in situ* or if they also render these cells to invade at a local site.

In the present study, we demonstrated that treatment of estradiol (E2), G1 (a GPR30 agonist) and tamoxifen (4-hydroxytamoxifen, OHT) induced phosphorylation of FAK at Y397 and cell migration in endometrial cancer cell lines. The mechanistic link and clinical relevance between GPR30 and FAK signaling were also demonstrated.

## Materials and Methods

### Patients and tissue specimens

Forty-nine patients who underwent surgery at Chang Gung Memorial Hospital (CGMH) and had pathological confirmation of endometrial cancer were included. Written informed consents were obtained from all participants. The study was approved by the Institutional Review Board of Chang Gung Memorial Hospital (CGMH-IRB#**98-2576B**).

### Cell culture and reagents

The RL95-2 cell line, derived from a well-differentiated adenocarcinoma of the endometrium [24], was obtained from the American Type Culture Collection. The Ishikawa cell line, a well-differentiated endometrial adenocarcinoma, is a gift from Dr. Nishida (Kasumigaura National Hospital, Japan) [25]. Ishikawa cells were maintained in minimum essential medium (α-MEM) containing 15% (v/v) fetal bovine serum (FBS) as well as 100 U/ml penicillin and 100 mg/ml streptomycin. RL95-2 cells were cultured in Dulbecco's minimum essential medium (DMEM/F12) with 10% fetal bovine serum (FBS; Biological industries, Israel), 100 U/ml penicillin and 100 mg/ml streptomycin. Both cells were incubated at 37°C in a humidified incubator with 5% CO2. The cells were grown to 80% confluence and transferred to serum-free medium for 24 h before treatment with 17β-estradiol (E2), G1 and 4-hydroxytamoxifen (OHT) and medroxyprogesterone acetate (MPA). E2, OHT, G1, MPA (Sigma), lapatinib (an EGFR inhibitor, GlaxoSmithKline plc, London, UK), FAK inhibitor 14 (Tocris Bioscience, UK) and SRC inhibitor 1 (sigma) were dissolved in dimethyl sulfoxide (DMSO).

### Phosphorylation-Kinase array

A phosphorylation-kinase array (Proteome Profiler™ Array kit, R&D systems) was used to search for specific phosphorylation kinases involved in the E2-induced signaling pathway. Before assay, RL95-2 cells were treated with E2 (1 μM) or DMSO (control) for 5 minutes followed by solubilization with lysis buffer (provided by the kit). The resulting protein lysates (500 μg) were incubated with nitrocellulose membranes containing 46 kinase phosphorylation sites at 4°C overnight followed by probing with detection antibodies and Streptavidin-HRP. The signals were detected by using an enhanced chemiluminescence (ECL) kit (Millipore). Densitometric analysis was performed by using UN-SCAN-IT software (Silk Scientific).

### Immunoblot analysis

The procedures of western blot analysis were previously reported [26], [27]. Cells were lysed in a buffer containing 20 mM Tris, pH 7.4, 2 mM EGTA, 2 mM Na_2_VO_3_, 2 mM Na_4_P_2_O_7_, 2% Triton X-100, 2% SDS, 1 μM aprotinin, 1 μM leupeptin and 1 mM PMSF. The protein concentration was analyzed by a protein assay kit with BSA standards according to the manufacturer's instructions (Bio-Rad Laboratories, Hercules, CA). Equal amounts of cell lysate were separated by SDS polyacrylamide gel electrophoresis (PAGE) and transferred to a nitrocellulose membrane (Hybond-C, Amersham Pharmacia Biotech Inc., Oakville, ON). Following blocking with Tris-buffered saline (TBS) containing 5% non-fat dry milk for 1 h, membranes were incubated overnight at 4°C with antibodies against ERα (epitomics), GPR30 (Genetex), FAK (Cell signaling), phosphorylated FAK at Y397 (pY397-FAK) (Abcam), phosphorylated FAK at 576/577 (pFAK-576/577) (Cell signaling), phospho-ERK1/2 (Santa Cruz), ERK1/2 (Santa Cruz), phosphorylated Src at Y416 (pSrc-416) (Cell signaling), Src inhibitor 1 (sigma) and β-actin (Sigma), followed by incubation with corresponding HRP-conjugated secondary antibodies. Immunoreactive bands were detected by using an enhanced chemiluminescence (ECL) kit (Millipore).

### Migration assay

Cell migration was evaluated by using a chemotaxis chamber (Corning) as previously reported [28], [29]. Cells (1×10^5^) in 200 μl of culture medium were applied to the upper chamber of the device, and 800 μl of medium containing 5 μg/ml fibronectin was added to the lower chamber. A polycarbonate membrane with a pore size of 8 μm was placed in between the two chambers. After 6 h of incubation at 37°C, the membrane was fixed in methanol for 10 min and stained in 20:2 water/Giemsa solution (Sigma) for 1 h. Migrated cells on the membrane were counted under a microscope.

### Immunohistochemistry of clinical samples

The procedures of immunohistochemical staining were reported previously [30]. The 4μm thick slides of formalin-fixed, paraffin-embedded tissues sectioned were deparaffinized in xylene and re-hydrated with a series of graded ethanol. Sections were then stained with an anti-human GPR30 polyclonal antibody (Genetex), an anti-human ERα monoclonal antibody (epitomics) or an anti-pFAK at Y397 monoclonal antibody (Invitrogen), using an automated IHC stainer with the Ventana Basic DAB Detection kit (Tucson, AZ, USA) according to the manufacturer's protocol. Counterstaining was performed with hematoxylin. The expression levels of ERα, GPR30 and pFAK in the endometrial cancer tissues were reviewed and scored by a pathologist. Histoscore (0–300) was obtained by the score (0–3) of intensity multiplying the percentage (0–100) of cells stained.

### GPR30 shRNA construction and DNA transfection

The shRNA sequences of GPR30 used in the present study were reported previously [31]. Two oligonuclotides, shGPR30F: ATCCCCCGCT -CCCTGCAAGCAGTCTTTTTCAAGAGAAAAGACTGCTTGCAGGGAGCG -TTTTTA and shGPR30R: AGCTTAAAAACGCTCCCTGCAAGCAGTCTTTT -CTCTTGAAAAAGACTGCTTGCAGGGAGCGGGG were anneled in a buffer containing100mM potassium acetate, 30 mM Hepes-KOH and 2 mM Magnesium acctate, and ligated with Hind III/Bgl II/CIP treated pSuper-neo vector (OligoEngine). Sequence was verified by autosequencing.

Procedures of DNA transfection were performed as previously described [32]. Briefly, RL95-2 cells were trypsined and re-suspended in serum free RPMI at the concentration of 10^7^ cells/ml. Cell suspensions (200 μl) were mixed with 10 μg shGPR30 plasmid and transferred to an electroporation cuvette with a 2 mm gap. Then cells were pulsed at 120 Voltage for 70 milliseconds in an electroporator (BTX ECM2001, Harvard Apparatus, Inc., Holliston, MA, USA). Thereafter, cells were re-seeded into six-well plates and maintained in DMEM/F12 with 10% fetal bovine serum overnight. On the following day, cells were starved in serum free medium for 24 hours, and then treated E2, G1 and OHT.

### Transfection of small interfering (si) RNA

Ishikawa cell (3x10^5^ cells in 6-well plates) was transfected with 50 nM of double-stranded RNA in Lipofectamine RNAimax (Invitrogen, Carlsbad, CA) according to manufacturer's protocol. Small interfering (si)-RNAs for GPR30 and EGFR were purchased from Invitrogen (Invitrogen, Carlsbad, CA). After 72 h of transfection, suppression of targeted genes was confirmed by RT-QPCR and western blot analyses.

The sequence of GPR30 siRNA used in the present study was: 5′-UCCUGUGCACCUUCAUGUCGCUCTT-3′ (sense).

The sequence for EGFR siRNA was: 5′-GCAGUCUUAUCUAACUAUGAUGCAA-3′ (sense).

The sequence for ERα siRNA was: 5′-AUCAGGUGGAUCAAAGUGUCUGUGA-3′ (sense).

## Results

### Estrogen induced phosphorylation of FAK at tyrosine 397 in RL95-2 endometrial cancer cells

**Figure 1:**
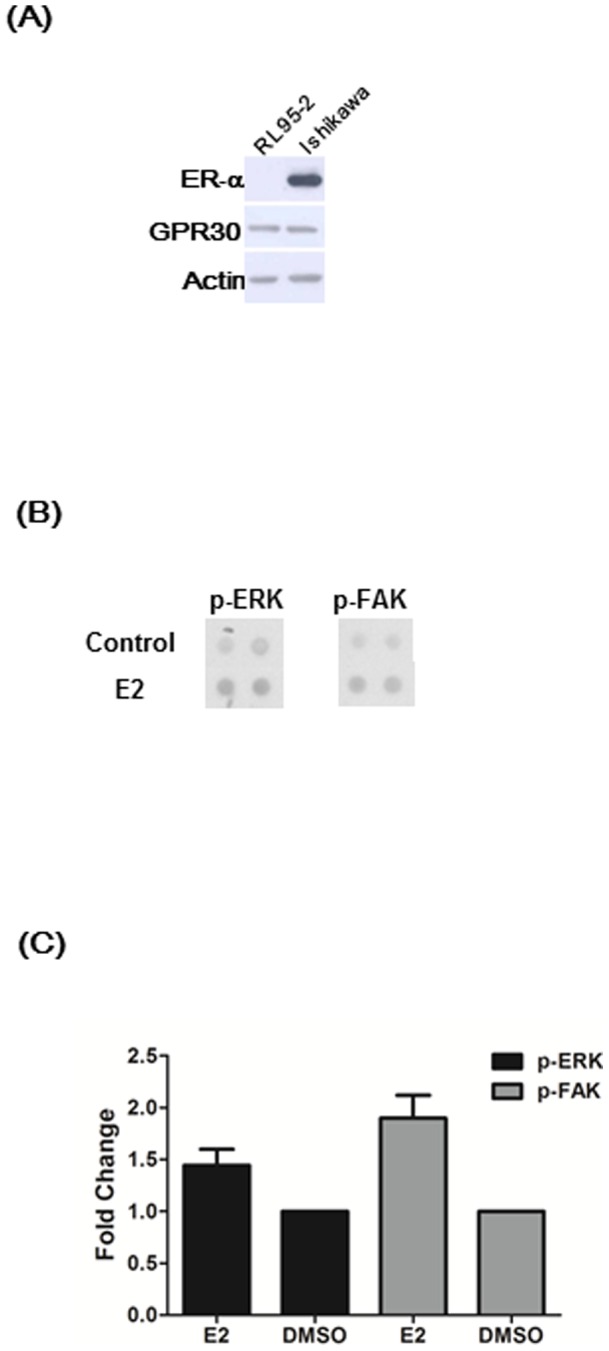
Treatment with 17β-estradiol (E2) activated phosphorylation of ERK and FAK in RL95-2 endometrial cancer cells. (**A**) ERα and GPR30 expression in endometrial cancer cells. In RL95-2 cells, GPR30 was highly expressed where ERα was not expressed after several cell passages with estrogen deprivation. In contrast, Ishikawa cells expressed both ERα and GPR30. (**B**) Phosphorylation of both ERK and FAK was increased after treatment of ERα-negative RL95-2 cells with E2 (1 μM) by using a set of phosphorylation arrays. (**C**) Fold changes of phosphorylation of ERK and FAK on the phosphorylation arrays (B) are shown. DMSO was used as control in comparison with E2 effects. Results shown were obtained from three independent experiments and are presented as mean ± standard deviation (SD).

An ERα-negative, GPR30-positive RL95-2 endometrial cancer cell line was established by depriving RL95-2 cells of estrogen through several passages (**Fig. 1A**). Using a set of phosphor-kinase arrays, treatment of ERα-negative RL95-2 endometrial cancer cells with E2 for 5 minutes significantly induced phosphorylation of ERK1/2 and of FAK at Y397 (**Fig. 1B**). Quantitatively, the levels of phosphorylated ERK1/2 and FAK were up-regulated by 1.4 and 1.9 folds, respectively, after E2 treatment as compared to control (DMSO) in RL95-2 cells (**Fig. 1C**).

### Estrogen induced phosphorylation of ERK and FAK at Y397 via GPR30 in endometrial cancer cells with or without nuclear ERα

**Figure 2:**
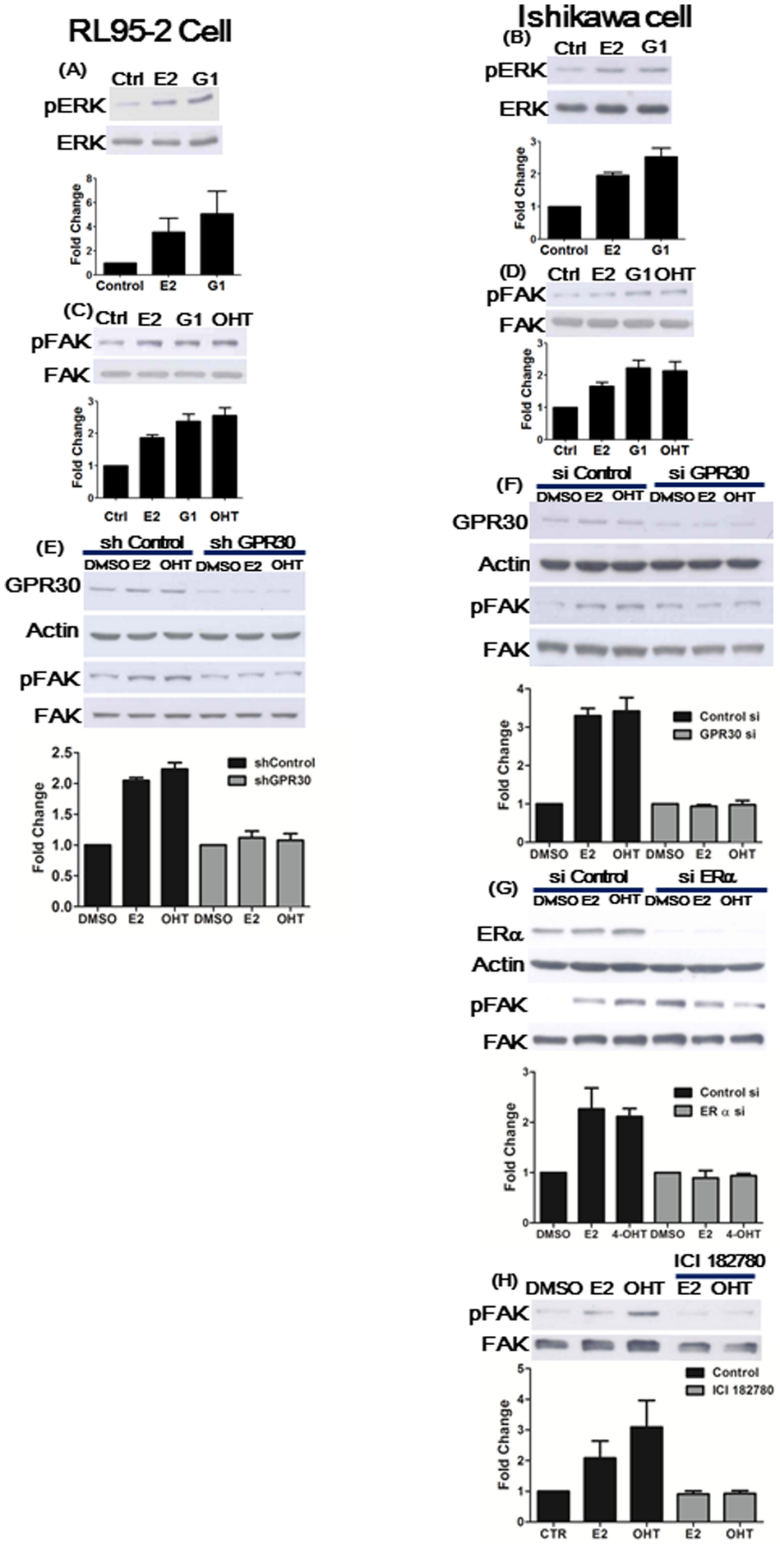
17β-estradiol (E2), GPR30 agonist (G1) and hydroxytamoxifen (OHT) triggered FAK Y397 phosphorylation through GPR30. RL95-2 (**A, C, E**) and Ishikawa cells (**B, D, F**) were treated with 1 μM E2, G1 and OHT for 5 mins. Phosphorylated ERK1/2 (pERK) (**A, B**) and FAK at Y397 (pFAK) (**C, D**) were detected by immunoblotting. Increased levels of pERK and pFAK were shown following treatment with E2, G1 and OHT as compared to control (DMSO). Endogenous GPR30 was knocked-down by shRNA in RL95-2 (**E**) or by siRNA in Ishikawa cells (**F**). Following treatment with 1 μM E2 and OHT for 5 mins, levels of pFAK were repressed in both endometrial cancer cells with knockdown of GPR30 (**E, F**). (**G**) In Ishikawa cells, knockdown of ERα using siRNA partially abolished phosphorylation of FAK induced by E2 and OHT. Similarly, E2- and OHT-induced phosphorylation of FAK was also blocked in the presence of ICI 182780 (an antagonist of ERα) (**H**). Results shown were obtained from three independent experiments and are presented as mean ± standard deviation (SD).

In ERα-negative RL95-2 cells, the expression levels of phosphorylated ERK and phosphorylated FAK at Y397 substantially increased after treatment of either E2, G1 (a GPR30 agonist) or tamoxifen (OHT, ERα antagonist) for 5 minutes (**Figs. 2A and 2C**). Similar results were also observed in Ishikawa endometrial cancer cells which were both ERα-positive and GPR30-positive (**Figs. 2B and 2D**). Using a specific shRNA or siRNA targeting GPR30, the endogenous expression of GPR30 was suppressed in both RL95-2 and Ishikawa cells (**Figs. 2E and 2F**). Subsequently, E2-induced phosphorylation of FAK at Y397 were abolished in cells with GPR30 knockdown (**Figs. 2E and 2F**). These findings suggest that E2-induced FAK phosphorylation at Y397 was mediated through GPR30 (the non-genomic estrogenic signaling pathway) in GPR30-positive endometrial cancer cells with or without the nuclear ERα receptor. However, knockdown of ERα using siRNA partially abolished phosphorylation of FAK induced by E2 and OHT in Ishikawa cells (GPR30-positive and ERα-positive) (**Fig. 2G**). Similarly, E2- and OHT-induced phosphorylation of FAK was also blocked in the presence of ICI 182780 (an antagonist of ERα) (**Fig. 2H**). Collectively, E2 and OHT may induce phosphorylation of FAK through both GPR30 and nuclear ERα.

### Estrogen induced cell migration was through GPR30 and FAK signaling in endometrial cancer cells

**Figure 3:**
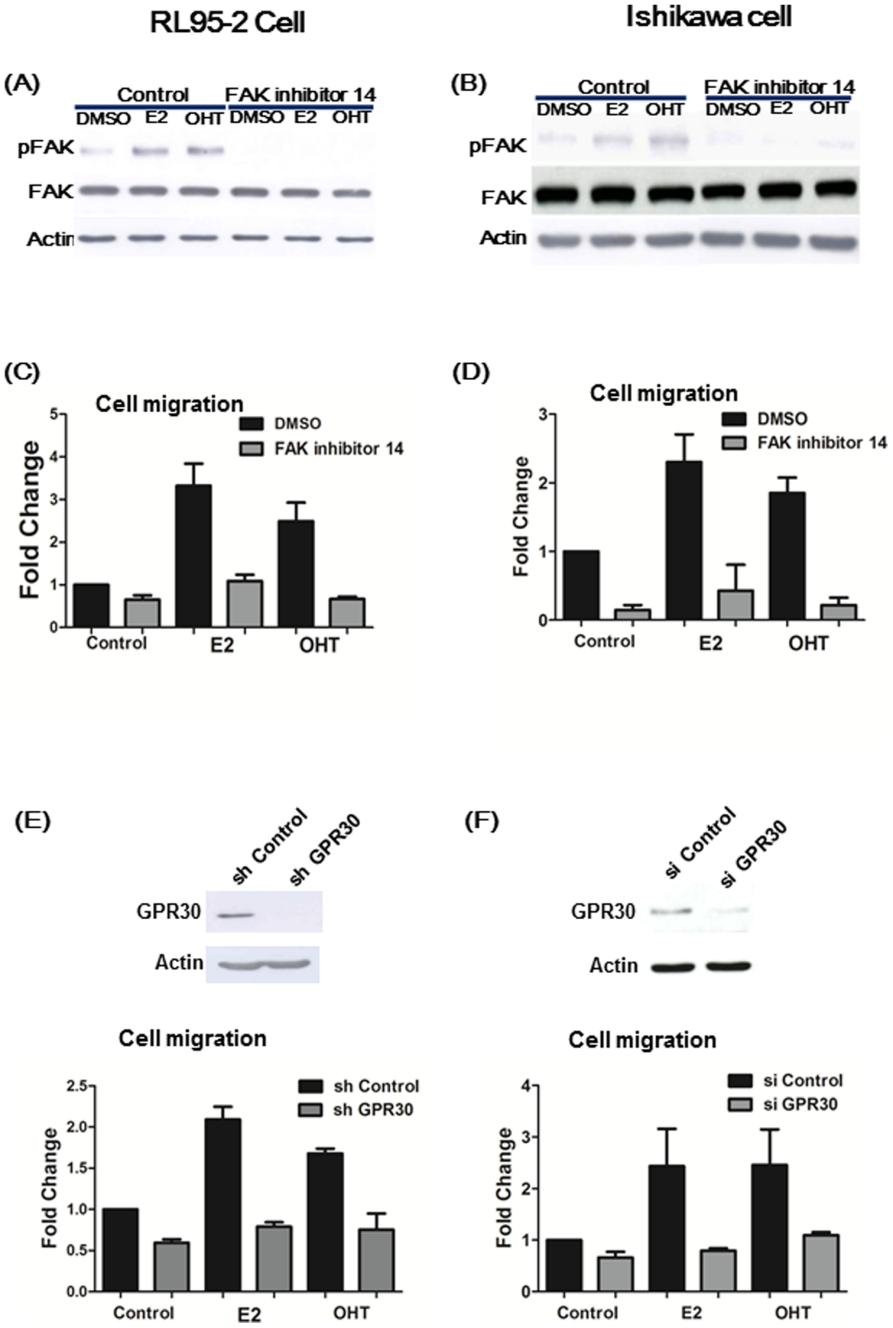
Cell migration induced by 17β-estradiol (E2) and tamoxifen (OHT) is mediated through GPR30 and phosphorylation of FAK at Y397 (pFAK) in endometrial cancer cells. RL95-2 cells (**A**) and Ishikawa cells (**B**) were pre-treated with FAK inhibitor 14 (15 μM) for 2 hrs, then treated with 1 μM E2 or OHT for 5mins, and cell lysates were analyzed via Western blot using antibody against pFAK Y397. The expression levels of pFAK at Y397 induced by E2 or OHT were suppressed by FAK inhibitor14. E2- and OHT- induced cell migration were also abrogated in the presence of FAK inhibitor 14 in RL95-2 (**C**) and Ishikawa cells (**D**). After knockdown of endogenous GPR30 in RL95-2 cells (**E**) and Ishikawa cells (**F**), cell migration was inhibited as compared with mock depletion. Results shown were obtained from three independent experiments and are presented as mean ± standard deviation (SD).

Since FAK has been reported to act as a tyrosine kinase and play a role in the invasion of tumor cells [33], we further examined the migration of RL95-2 and Ishikawa cells through GPR30-FAK pathway in the presence of E2 or tamoxifen (OHT) by transwell migration assay. To further dissect the role of phosphorylated FAK in cell migration, an FAK-specific inhibitor preventing FAK phosphorylation at Y397 (FAK inhibitor 14) [34] was used to analyze changes in the level of phosphorylated FAK at Y397 and its subsequent effect on cell migration. As shown in **Figs. 3A and 3B**, the phosphorylation of FAK at Y397 induced by E2 or OHT were blocked by FAK inhibitor 14. Treatment of E2 and OHT stimulated cell migration by 2.3 and 2.5 folds in RL95-2 cells, and by 2.3 and 1.9 folds in Ishikawa cells, respectively, as compared to the control (DMSO). In contrast, E2 or OHT-induced cell migration were abolished by the treatment with FAK inhibitor 14 in RL95-2 and Ishikawa cells (**Figs. 3C and 3D**). We also investigated the role of GPR30 in cell migration induced by E2 or OHT (**Fig. 3E and 3F**). The migration ability was induced by 2.1 folds (in RL95-2 cells) and 2.4 folds (in Ishikawa cells) in the presence of E2 in cells transfected with control shRNA, and the induction of cell migration after treatment with E2 was blocked in both cells with GPR30 knockdown. Similarly, tamoxifen (OHT) activated cell migration by 1.7 folds (in RL95-2 cells) and 2.4 folds (in Ishikawa cells), and these effects were inhibited in both cells with knockdown of GPR30. These results indicated that the induction of cell migration by estrogen and tamoxifen is mediated through GPR30 signaling and the phosphorylation of FAK at Y397 in endometrial cancer cells.

### E2, G1 and OHT induced FAK phosphorylation through EGFR-PI3K-ERK pathway in RL95-2 endometrial cancer cells

**Figure 4:**
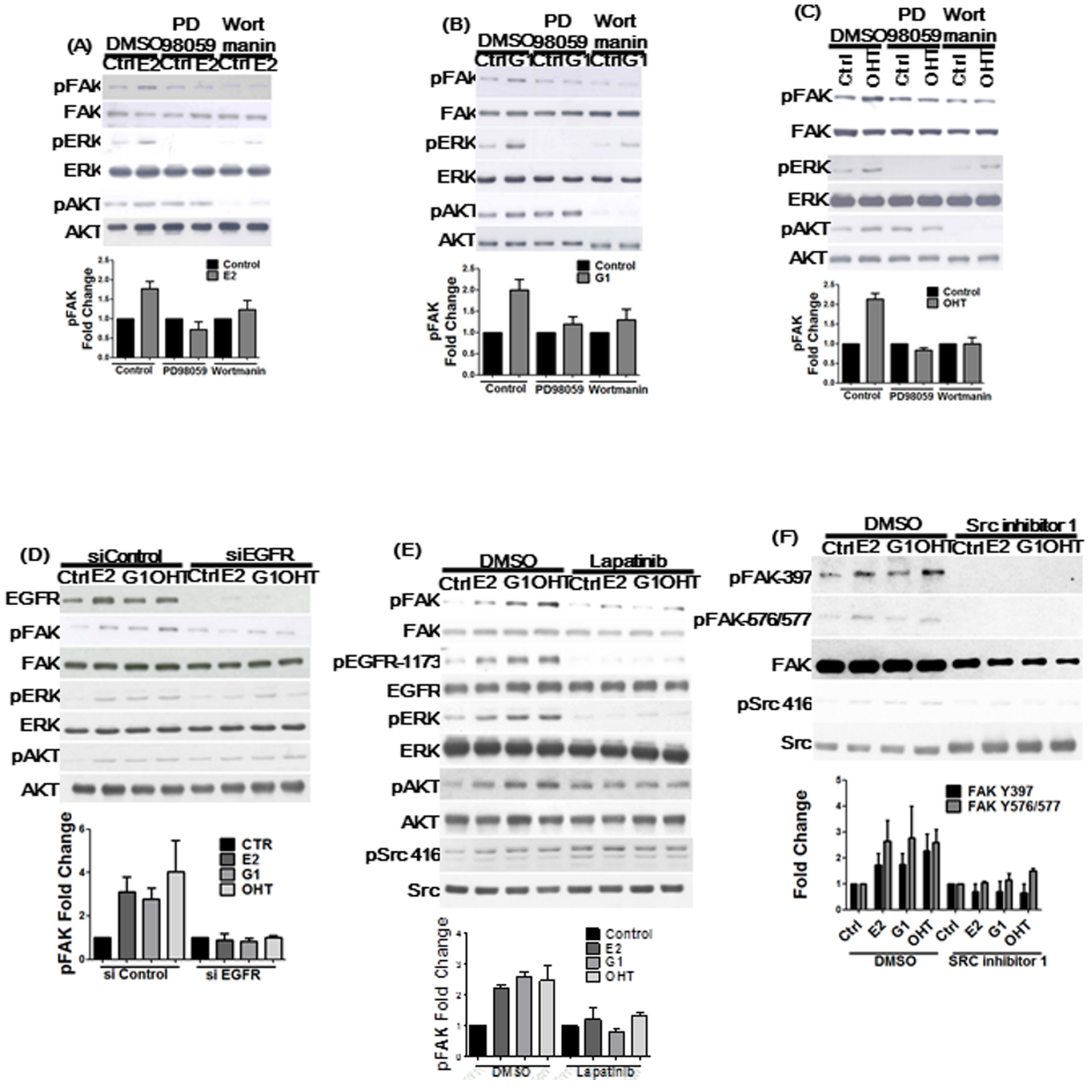
Inhibitors or siRNA of kinases suppressed E2-, G1- and OHT-induced phosphorylation of FAK in RL95-2 endometrial cancer cells. RL95-2 cells were pretreated with ERK inhibitor (PD98059, 100 μM) or a PI3K inhibitor (Wortmanin, 100 nM) for 1 hr followed by administration of 1 µM (**A**) E2, (**B**) G1 or (**C**) OHT for 5 mins. The ERK and PI3K inhibitors suppressed phosphorylation of FAK in the presence of E2, G1 or OHT. (**D**) The phosphorylation of FAK, AKT and ERK was repressed in RL95-2 cells with knockdown of EGFR using siRNA after treatment with 1 μM E2, G1 or OHT for 5 mins. (**E**) With an exception of Src, the phosphorylation of FAK, AKT, ERK and EGFR was abrogated in RL95-2 cells pre-treated with 120 nM lapatinib (an EGFR inhibitor) for 2 hrs followed by treatment with 1 μM E2, G1 or OHT for 5 mins. (**F**) In RL95-2 cells, E2-, G1- and OHT-induced phosphorylation of FAK at both 397 and 576/577 was repressed in the presence of Src inhibitor 1. Results shown were obtained from three independent experiments and are presented as mean ± standard deviation (SD).

GPR30 transactivates epidermal growth factor receptors (EGFRs) and in turn activates downstream signalings including mitogen-activated protein kinases (MAPK) and phosphatidylinositol 3-kinases (PI3K) pathways [2]. It is also evident that PI3K regulates FAK phosphorylation at Y397, the first step in FAK activation [35]. In order to verify that estrogen-induced GPR30 signaling is mediated through EGFR/PI3K/FAK pathway in endometrial cancer cells, we used specific inhibitors or siRNA to test this pathway. First, knockdown of EGFR expression by siRNA or blockage of its tyrosin kinase activity using lapatinib (an EGFR inhibitor) inhibited phosphorylation of FAK induced by E2, G1 or OHT in RL95-2 endometrial cancer cells (**Figs. 4D and 4E**), suggesting that estrogen-induced GPR30 signaling is mediated through EGFR. Moreover, phosphorylation of both AKT and ERK was also repressed after application of siRNA targeting EGFR or lapatinib (**Figs. 4D and 4E**), suggested that PI3K may be involved downstream of EGFR. Indeed, treatment of wortmanin, a PI3K inhibitor, blocked FAK phosphorylation at Y397 induced by E2, G1 or OHT in RL95-2 endometrial cancer cells. These findings further confirm previous reports that E2 activated GPR30 signaling pathway is through PI3K and ERK1/2 downstream signals [**36**]. Furthermore, repression of ERK activity by PD98059 (an ERK inhibitor) also prevented phosphorylation of FAK at Y397. Together, these findings demonstrated that E2-induced FAK phosphorylation is mediated through GPR30-EGFR-PI3K-ERK signaling pathway.

### The involvement of Src in the phosphorylation of FAK induced by E2, G1 and OHT in RL95-2 endometrial cancer cells

To further verify the involvement of Src in the phosphorylation of FAK, we pre-treated RL95-2 cells with 50 nM Src inhibitor 1 for 24 hrs followed by treatment with 1 μM E2, G1 or OHT for 5 mins. Consequently, E2-, G1- and OHT-induced phosphorylation of FAK at both 397 and 576/577 was repressed in the presence of Src inhibitor 1 (**Fig. 4F**), indicating that Src was involved in the GPR30-EGFR signaling pathway. However, unlike diminished phosphorylation of FAK by lapatinib (an EGFR inhibitor), the phosphorylation of Src was not abolished in the presence of lapatinib in RL95-2 cells (**Fig. 4E**), suggesting that the phosphorylation of Src might be concomitantly regulated by other signaling pathways rather than EGFR.

### The involvement of phosphorylation of FAK in cell proliferation in endometrial cancer cells

Previous reports have demonstrated that phosphorylation of FAK is involved in the cell proliferation [38] and that c-fos (a target gene of GPR30) also play a role in cell proliferation [11]. To verify the role of phosphorylation of FAK in regulation of c-fos, expression of c-fos mRNA induced by G1 (a GPR30 agonist) in the presence or absence of FAK inhibitor 14 was determined by Taqman real-time quantitative polymerase chain reaction (PCR) in endometrial cancer cells. Consequently, expression of c-fos mRNA induced by G1 was abolished in the presence of FAK inhibitor 14 in both RL95-2 and Ishikawa endometrial cancer cells (**Figure S1**). Using the BrdU incorportation assay, cell proliferation of RL95-2 induced by E2 or OHT was substantially repressed in presence of FAK inhibitor 14 (**Figure S2**). These findings indicated that phosphorylation of FAK played a role in regulation of c-fos and in turn the cell proliferation.

### Expression of GPR30 and phosphorylated FAK at Y397 in human endometrial cancer tissues

**Figure 5:**
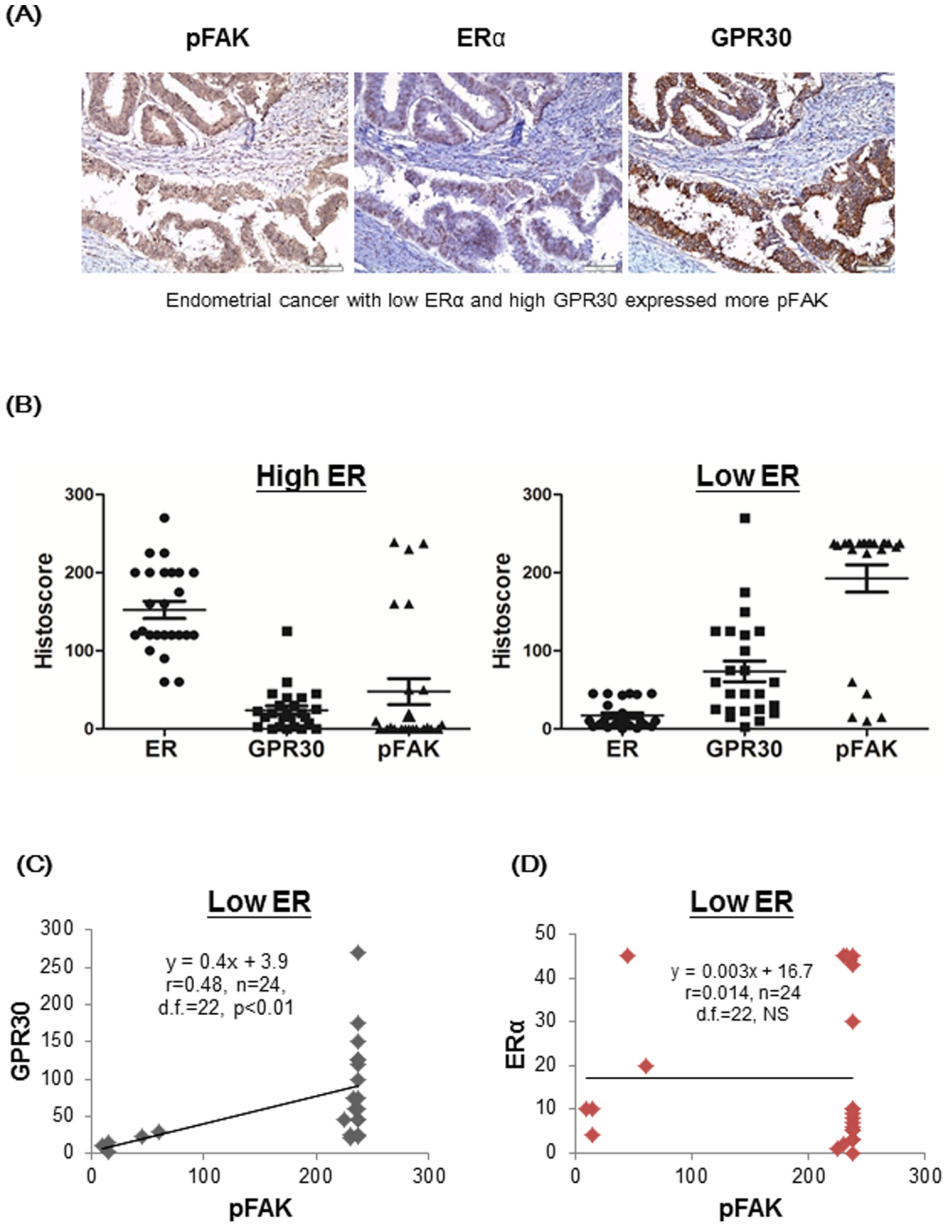
Expression of ERα, GPR30 and pFAK (at Y397) in human endometrial cancers with low ERα. (**A**) In clinical samples of human endometrial cancers with low expression of ERα (histoscore  = 10), relatively high expression of GPR30 (histoscore  = 270) and pFAK (histoscore  = 225) were observed (40× magnification of the object lens). (**B**) The histoscores of both GPR30 and pFAK were lower in human endometrial cancers with high ER (histoscore >45, left panel) as compared to those with low ER (histoscore ≤45, right panel). (**C**) There was a positive correlation of histoscores between pFAK and GPR30 in human endometrial cancers with low ERα. (**D**) No association was found in histoscores between pFAK and ERα in human endometrial cancers. NS: not significant.

Since our findings demonstrated that GPR30-mediated estrogen signaling leads to FAK phosphorylation and subsequent cell migration, we wish to further investigate the disease correlation of this pathway in clinical samples. To do so, we used immunohistochemical staining to detect and quantify the expression of ERα, GPR30 and pFAK in human endometrial cancers. As showed in **Figs. 5A and 5B**, human endometrial cancers (n = 24) with low expression of nuclear ERα (histoscore ≤45) were found to have significantly higher expression of both GPR30 and pFAK as compared to those (n = 25) with high ERα (histoscore >45) (p<0.0001) (**Table 1**). Clinically, cases with low expression of nuclear ERα studied reveal evidence of increased propensity for invasion (staging from IB to IVA). Moreover, level of phosphorylated FAK was significantly correlated with expression of GPR30 in human endometrial cancers with low ERα (p<0.05) (**Fig. 5C**), but no such correlation was detected between ERα and pFAK (**Fig. 5D**).

**Table 1:** Histoscores of estrogen receptor (ER), GPR30 and phosphorylated FAK (pFAK) in human endometrial cancers.

	Low ER	High ER	p value*
	(histoscore <45, n = 24)	(histoscore >45, n = 25)	
ER	17.1±17.3	152.4±54.6	<0.0001
GPR30	73.6±64.5	23.9±26.8	<0.0001
pFAK	192.6±86.3	47.8±83.3	<0.0001

Data are presented as mean ± standard deviation (SD).

* Student t-test.

## Discussion

GPR30 has been proposed to act as a steroid receptor localized either to the cell plasma membrane or to the endoplasmic reticulum and mediate rapid action of estrogen [21], [22]. It is also evident that GPR30 triggers cellular proliferation through activation of phosphokinases, e.g. phosphatidyinositol 3-kinase (PI3K) and mitogen-activated protein kinase (MAPK), in response to estrogen and tamoxifen [7], [8], [11]. Nevertheless, there are some reports demonstrating that estrogen acts independently of GPR30 in some specific cell types [39]–[42]. Notably, a study has shown that GPR30 is incapable of mediating estrogen action, such as failure of activating cAMP, ERK or PI3K following estrogen treatment, in endothelial cells without ERα and ERβ [41]. In the present study, knockdown of GPR30 in RL95-2 and Ishikawa endometrial cancer cells abrogated E2 and tamoxifen induced phosphorylation of FAK and abolished the subsequent cell migration (**Figs. 2E, 2F** **,** **3E and 3F**), indicating that GPR30 is capable of mediating estrogen action in endometrial cancer cells with or without nuclear ERα.

Tamoxifen is a selective estrogen receptor modulator (SERM), acting as an ERα antagonist in breast but a weak estrogen in uterine endometrium. Anti-cancer treatment with tamoxifen in breast cancer relates to an increased incidence of endometrial cancer [43]–[46]. Mechanistically, tamoxifen activates MAPK pathway through GPR30 to promote proliferation of endometrial cancer cells [47]. As with promotion of cell proliferation, tamoxifen activated the FAK pathway in both RL95-2 and Ishikawa endometrial cancer cells (**Figs. 3A, 3B, 3C and 3D**). In addition, results involving GPR30 depletion indicated that GPR30 was essential to promote tamoxifen-induced cell migration in endometrial cancers (**Figs. 3E and 3F**). Collectively, tamoxifen is capable of provoking cell migration via GPR30 in endometrial cancer cells with or without nuclear ERα.

There is evidence demonstrating that estrogen triggers epidermal growth factor receptor (EGFR) pathway through GPR30 in breast cancer cells [36]. Lapatinib is a tyrosin kinase inhibitor of EGFR and blocks EGFR downstream signaling in cancer cell [48]. In this study, lapatinib repressed the phosphorylation of FAK induced by both E2 and tamoxifen in endometrial cancer cells (**Fig. 4E**). In addition, knockdown of EGFR expression by siRNA also inhibited E2- and tamoxifen-induced phosphorylation of FAK in endometrial cancer cells (**Fig. 4D**). These findings demonstrated that EGFR is essential in the GPR30-mediated pathway and further support the notion that lapatinib, in combination with tamoxifen, may be used in patients with breast cancers for preventing the development of endometrial cancer [49].

Cell migration is a crucial attribute of cancer cells that influences tumor invasive potential into adjacent tissues. A better understanding of the mechanism of cell migration induced by estrogens is essential to the development of efficient therapies for endometrial cancer. In this regard, FAK has been recognized to localize to the plasma membrane at sites of focal adhesion complexes formation and acts as a key regulator in cell migration and cell invasion involving proteolytic degradation of the extracellular matrix [47]. Results from the present study also indicated that the FAK pathway played an important role in mediating cell migration induced by E2 and tamoxifen in RL95-2 and Ishikawa endometrial cancer cells (**Figs. 3A, 3B, 3C and 3D**). In addition, studies using inhibitors and siRNA also demonstrated that FAK-dependent cell migration is mediated through EGFR, PI3K and ERK (**Figs. 4A, 4B, 4C, 4D and 4E**). Collectively, our data suggested that estrogen-induced cell migration is mediated through the activation of PI3K/ERK/FAK pathway in endometrial cancer cells with low or without nuclear ERα.

Noteworthily, it is also possible that estrogen-induced cell migration is mediated through connective tissue growth factor (CTGF). A previous report has shown that CTGF is a GPR30 target gene and GPR30 signaling promotes cell migration through CTGF induction in ER-negative human breast cancer cells [37]. This pathway needs to be evaluate to further elucidate the mechanism of estrogen-induced cell migration in endometrial cancer cells.

Pathologically, type 1 human endometrial cancers are estrogen-sensitive and have good prognosis, whereas type 2 human endometrial cancers are not associated with increased exposure to estrogen and carry poorer prognosis [50]. In the present study, human endometrial cancers (n = 24) with low ERα were found to express higher levels of GPR30 and pFAK as compared to those (n = 25) with high ERα (**Fig. 5B** and **Table 1**). Additionally, GPR30 and pFAK were positively correlated in human endometrial cancers with low ERα, but no such correlation was found between ERα and pFAK (**Figs. 5C and 5D**). These results from the *in vivo* studies indicated that endometrial cancers with low ERα might be extremely sensitive to estrogen. Consequently, these endometrial cancers with low ERα might express higher levels of GPR30 and pFAK even with low circulating serum levels of estrogen and without the addition of exogenous estrogen, which implies that they have a potential to invade adjacent tissues. Moreover, we also confirmed previous reports demonstrating that E2 and OHT trigger rapid ERK and FAK signaling pathway and induce cell motility in endometrial cancer cell [51]. Collectively, our cell culture results and correlations observed in the clinical samples imply that *in vivo* estrogen-induced phosphorylation of FAK is mediated through GPR30 rather than nuclear ERα in human endometrial cancers with low ERα.

**Figure 6:**
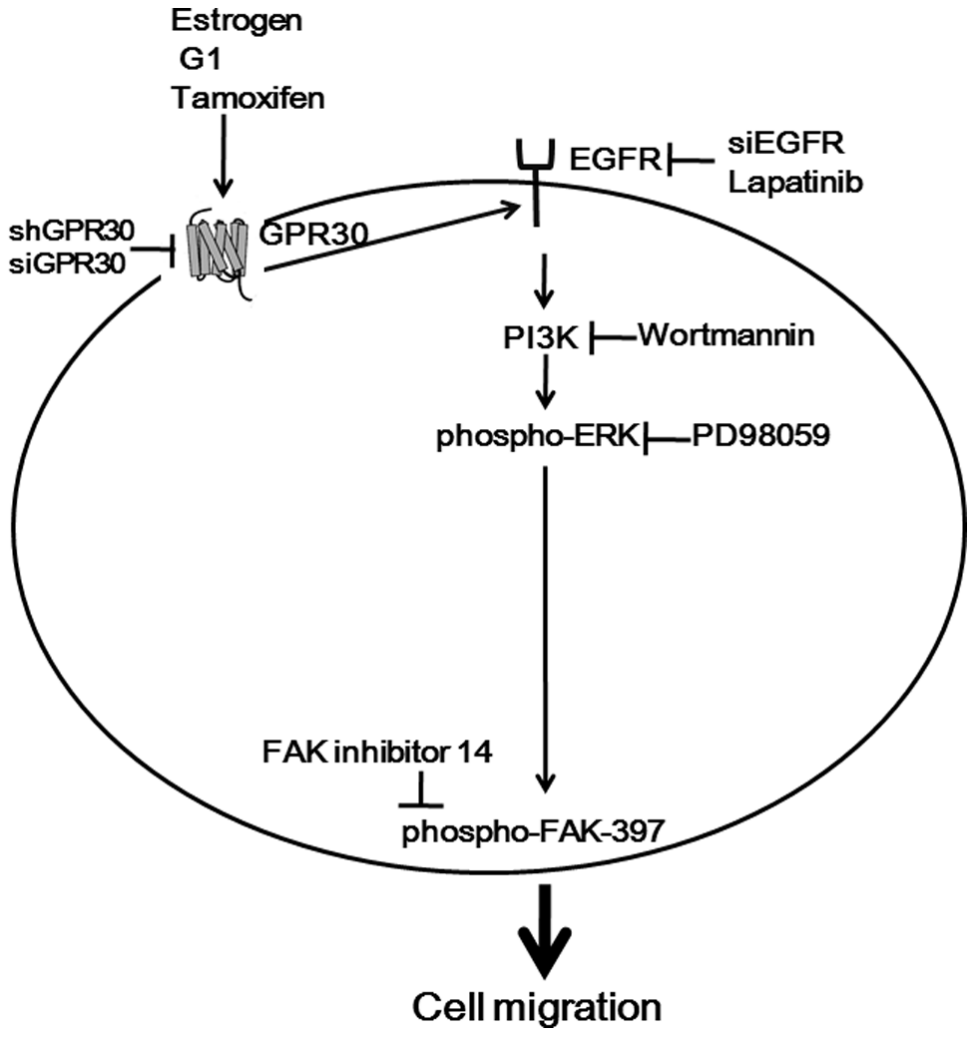
E2, G1 and OHT promoted cell migration through the GPR30/EGFR/PI3K/ERK/FAK signaling pathway. Our current model of activation of the GPR30-EGFR-PI3K-ERK-FAK pathway by E2, G1 and OHT.

In conclusion, we demonstrated for the first time that estrogen- and antiestrogen-induced migration of endometrial cancer cells was through GPR30 signaling and activation of EGFR/PI3K/ERK/FAK pathway in endometrial cancer cells (**Fig. 6**). Our findings may provide new insights regarding the mechanisms of estrogen- and antiestrogen-induced cell migration in endometrial cancer cells with low or without ERs, suggesting the possibility of anti-GPR30 as a potential therapeutic intervention for the treatment of human endometrial cancers.

## Supporting Information

Figure S1 **Expression of c-fos mRNA induced by G1 (a GPR30 agonist) in endometrial cancer cell lines (RL95-2 and Ishikawa) in the absence or presence of FAK inhibitor 14.** To evaluate whether the activation of FAK play a role in the regulation of GPR30 target genes, endometrial cancer cells were pre-treated with FAK inhibitor 14 (15 μM) for 2 hr followed by administration of 1 µM G1 for 1 hr. Then, mRNA of c-fos in endometrial cancer cells was determined by Taqman real-time quantitative polymerase chain reaction (PCR). Expression of c-fos mRNA induced by G1 was abolished in the presence of FAK inhibitor 14 in both RL95-2 and Ishikawa endometrial cancer cells. Results shown were obtained from three independent experiments and are presented as mean + standard deviation (SD).
(PDF)

Figure S2 **FAK inhibitor 14 blocked cell proliferation induced by 17β-estradiol (E2) and 4-hydroxytamoxifen (OHT) in ERα-negative endometrial cell RL95-2.** BrdU incorportation assay was used as an index for cell proliferation. RL95-2 cells were treated with E2 or OHT (1 μM) in presence or absence of FAK inhibitor 14 (1 μM) for 48 hr. Consequently, cell proliferation of RL95-2 induced by E2 or OHT was substantially repressed in presence of FAK inhibitor 14. Results shown were obtained from three independent experiments and are presented as mean + standard deviation (SD).
(PDF)

## References

[1] Kovacs EJ, Messingham KA, Gregory MS (2002) Estrogen regulation of immune responses after injury. Mol Cell Endocrinol 193: 129–135. 12161012 10.1016/s0303-7207(02)00106-5

[2] Prossnitz ER, Arterburn JB, Smith HO, Oprea TI, Sklar LA, et al (2008) Estrogen signaling through the transmembrane G protein-coupled receptor GPR30. Annu Rev Physiol 70: 165–190. 18271749 10.1146/annurev.physiol.70.113006.100518

[3] Strom A, Hartman J, Foster JS, Kietz S, Wimalasena J, et al (2004) Estrogen receptor beta inhibits 17beta-estradiol-stimulated proliferation of the breast cancer cell line T47D. Proc Natl Acad Sci U S A 101: 1566–1571. 14745018 10.1073/pnas.0308319100 PMC341775

[4] Falkenstein E, Tillmann HC, Christ M, Feuring M, Wehling M (2000) Multiple actions of steroid hormones – a focus on rapid, nongenomic effects. Pharmacol Rev 52: 513–556. 11121509

[5] Filardo EJ, Thomas P (2005) GPR30: a seven-transmembrane-spanning estrogen receptor that triggers EGF release. Trends Endocrinol Metab 16: 362–367. 16125968 10.1016/j.tem.2005.08.005

[6] Filardo EJ, Quinn JA, Bland KI, Frackelton AR Jr (2000) Estrogen-induced activation of Erk-1 and Erk-2 requires the G protein-coupled receptor homolog, GPR30, and occurs via trans-activation of the epidermal growth factor receptor through release of HB-EGF. Mol Endocrinol 14: 1649–1660. 11043579 10.1210/mend.14.10.0532

[7] Revankar CM, Cimino DF, Sklar LA, Arterburn JB, Prossnitz ER (2005) A transmembrane intracellular estrogen receptor mediates rapid cell signaling. Science 307: 1625–1630. 15705806 10.1126/science.1106943

[8] Thomas P, Pang Y, Filardo EJ, Dong J (2005) Identity of an estrogen membrane receptor coupled to a G protein in human breast cancer cells. Endocrinology 146: 624–632. 15539556 10.1210/en.2004-1064

[9] Isensee J, Meoli L, Zazzu V, Nabzdyk C, Witt H, et al (2009) Expression pattern of G protein-coupled receptor 30 in LacZ reporter mice. Endocrinology 150: 1722–1730. 19095739 10.1210/en.2008-1488

[10] Olde B, Leeb-Lundberg LM (2009) GPR30/GPER1: searching for a role in estrogen physiology. Trends Endocrinol Metab 20: 409–416. 19734054 10.1016/j.tem.2009.04.006

[11] Maggiolini M, Vivacqua A, Fasanella G, Recchia AG, Sisci D, et al (2004) The G protein-coupled receptor GPR30 mediates c-fos up-regulation by 17beta-estradiol and phytoestrogens in breast cancer cells. J Biol Chem 279: 27008–27016. 15090535 10.1074/jbc.M403588200

[12] Prossnitz ER, Maggiolini M (2009) Mechanisms of estrogen signaling and gene expression via GPR30. Mol Cell Endocrinol 308: 32–38. 19464786 10.1016/j.mce.2009.03.026 PMC2847286

[13] Vivacqua A, Bonofiglio D, Albanito L, Madeo A, Rago V, et al (2006) 17beta-estradiol, genistein, and 4-hydroxytamoxifen induce the proliferation of thyroid cancer cells through the g protein-coupled receptor GPR30. Mol Pharmacol 70: 1414–1423. 16835357 10.1124/mol.106.026344

[14] Smith HO, Leslie KK, Singh M, Qualls CR, Revankar CM, et al.. (2007) GPR30: a novel indicator of poor survival for endometrial carcinoma. Am J Obstet Gynecol 196: 386 e381–389; discussion 386 e389–311. 10.1016/j.ajog.2007.01.004 17403429

[15] Smith HO, Arias-Pulido H, Kuo DY, Howard T, Qualls CR, et al (2009) GPR30 predicts poor survival for ovarian cancer. Gynecol Oncol 114: 465–471. 19501895 10.1016/j.ygyno.2009.05.015 PMC2921775

[16] Ignatov T, Eggemann H, Semczuk A, Smith B, Bischoff J, et al.. (2010) Role of GPR30 in endometrial pathology after tamoxifen for breast cancer. Am J Obstet Gynecol 203: 595 e599–516. 10.1016/j.ajog.2010.07.034 20965484

[17] Mitra SK, Hanson DA, Schlaepfer DD (2005) Focal adhesion kinase: in command and control of cell motility. Nat Rev Mol Cell Biol 6: 56–68. 15688067 10.1038/nrm1549

[18] Robles E, Gomez TM (2006) Focal adhesion kinase signaling at sites of integrin-mediated adhesion controls axon pathfinding. Nat Neurosci 9: 1274–1283. 16964253 10.1038/nn1762

[19] van Nimwegen MJ, van de Water B (2007) Focal adhesion kinase: a potential target in cancer therapy. Biochem Pharmacol 73: 597–609. 16997283 10.1016/j.bcp.2006.08.011

[20] Miyazaki T, Kato H, Nakajima M, Sohda M, Fukai Y, et al (2003) FAK overexpression is correlated with tumour invasiveness and lymph node metastasis in oesophageal squamous cell carcinoma. Br J Cancer 89: 140–145. 12838315 10.1038/sj.bjc.6601050 PMC2394235

[21] Creasman WT (2002) Estrogen and cancer. Gynecol Oncol 86: 1–9. 12079291 10.1006/gyno.2001.6499

[22] Lepine J, Audet-Walsh E, Gregoire J, Tetu B, Plante M, et al (2010) Circulating estrogens in endometrial cancer cases and their relationship with tissular expression of key estrogen biosynthesis and metabolic pathways. J Clin Endocrinol Metab 95: 2689–2698. 20371658 10.1210/jc.2010-2648

[23] Flamini MI, Sanchez AM, Genazzani AR, Simoncini T (2011) Estrogen regulates endometrial cell cytoskeletal remodeling and motility via focal adhesion kinase. Fertil Steril 95: 722–726. 20869705 10.1016/j.fertnstert.2010.08.039

[24] Way DL, Grosso DS, Davis JR, Surwit EA, Christian CD (1983) Characterization of a new human endometrial carcinoma (RL95-2) established in tissue culture. In Vitro 19: 147–158. 6339371 10.1007/BF02618053

[25] Nishida M, Kasahara K, Kaneko M, Iwasaki H, Hayashi K (1985) [Establishment of a new human endometrial adenocarcinoma cell line, Ishikawa cells, containing estrogen and progesterone receptors]. Nippon Sanka Fujinka Gakkai Zasshi 37: 1103–1111. 4031568

[26] Wang TH, Chao AS, Chen JK, Chao A, Chang YL, et al (2009) Network analyses of differentially expressed proteins in amniotic fluid supernatant associated with abnormal human karyotypes. Fertil Steril 92: 96–107. 19108832 10.1016/j.fertnstert.2008.05.038

[27] Wang TH, Chao A, Tsai CL, Chang CL, Chen SH, et al (2010) Stress-induced phosphoprotein 1 as a secreted biomarker for human ovarian cancer promotes cancer cell proliferation. Mol Cell Proteomics 9: 1873–1884. 20501939 10.1074/mcp.M110.000802 PMC2938116

[28] Chao A, Lin CY, Lee YS, Tsai CL, Wei PC, et al (2012) Regulation of ovarian cancer progression by microRNA-187 through targeting Disabled homolog-2. Oncogene 31: 764–775. 21725366 10.1038/onc.2011.269

[29] Tsai CL, Tsai CN, Lin CY, Chen HW, Lee YS, et al (2012) Secreted stress-induced phosphoprotein 1 activates the ALK2-SMAD signaling pathways and promotes cell proliferation of ovarian cancer cells. Cell Rep 2: 283–293. 22884369 10.1016/j.celrep.2012.07.002

[30] Chao A, Wang TH, Lee YS, Hsueh S, Chao AS, et al (2006) Molecular characterization of adenocarcinoma and squamous carcinoma of the uterine cervix using microarray analysis of gene expression. Int J Cancer 119: 91–98. 16450401 10.1002/ijc.21813

[31] Albanito L, Sisci D, Aquila S, Brunelli E, Vivacqua A, et al (2008) Epidermal growth factor induces G protein-coupled receptor 30 expression in estrogen receptor-negative breast cancer cells. Endocrinology 149: 3799–3808. 18467441 10.1210/en.2008-0117 PMC2488235

[32] Chao A, Tsai CL, Wei PC, Hsueh S, Chao AS, et al (2010) Decreased expression of microRNA-199b increases protein levels of SET (protein phosphatase 2A inhibitor) in human choriocarcinoma. Cancer Lett 291: 99–107. 19900756 10.1016/j.canlet.2009.10.005

[33] Siesser PM, Hanks SK (2006) The signaling and biological implications of FAK overexpression in cancer. Clin Cancer Res 12: 3233–3237. 16740741 10.1158/1078-0432.CCR-06-0456

[34] Golubovskaya VM, Nyberg C, Zheng M, Kweh F, Magis A, et al (2008) A small molecule inhibitor, 1,2,4,5-benzenetetraamine tetrahydrochloride, targeting the y397 site of focal adhesion kinase decreases tumor growth. J Med Chem 51: 7405–7416. 18989950 10.1021/jm800483v PMC2662449

[35] Thamilselvan V, Craig DH, Basson MD (2007) FAK association with multiple signal proteins mediates pressure-induced colon cancer cell adhesion via a Src-dependent PI3K/Akt pathway. FASEB J 21: 1730–1741. 17317726 10.1096/fj.06-6545com

[36] Filardo EJ (2002) Epidermal growth factor receptor (EGFR) transactivation by estrogen via the G-protein-coupled receptor, GPR30: a novel signaling pathway with potential significance for breast cancer. J Steroid Biochem Mol Biol 80: 231–238. 11897506 10.1016/s0960-0760(01)00190-x

[37] Pandey DP, Lappano R, Albanito L, Madeo A, Maggiolini M, et al (2009) Estrogenic GPR30 signalling induces proliferation and migration of breast cancer cells through CTGF. EMBO J 28: 523–532. 19153601 10.1038/emboj.2008.304 PMC2657575

[38] Pirone DM, Liu WF, Ruiz SA, Gao L, Raghavan S, et al (2006) An inhibitory role for FAK in regulating proliferation: a link between limited adhesion and RhoA-ROCK signaling. J Cell Biol 174: 277–288. 16847103 10.1083/jcb.200510062 PMC2064187

[39] Otto C, Rohde-Schulz B, Schwarz G, Fuchs I, Klewer M, et al (2008) G protein-coupled receptor 30 localizes to the endoplasmic reticulum and is not activated by estradiol. Endocrinology 149: 4846–4856. 18566127 10.1210/en.2008-0269

[40] Madak-Erdogan Z, Kieser KJ, Kim SH, Komm B, Katzenellenbogen JA, et al (2008) Nuclear and extranuclear pathway inputs in the regulation of global gene expression by estrogen receptors. Mol Endocrinol 22: 2116–2127. 18617595 10.1210/me.2008-0059 PMC2631368

[41] Pedram A, Razandi M, Levin ER (2006) Nature of functional estrogen receptors at the plasma membrane. Mol Endocrinol 20: 1996–2009. 16645038 10.1210/me.2005-0525

[42] Ahola TM, Manninen T, Alkio N, Ylikomi T (2002) G protein-coupled receptor 30 is critical for a progestin-induced growth inhibition in MCF-7 breast cancer cells. Endocrinology 143: 3376–3384. 12193550 10.1210/en.2001-211445

[43] Swerdlow AJ, Jones ME (2005) Tamoxifen treatment for breast cancer and risk of endometrial cancer: a case-control study. J Natl Cancer Inst 97: 375–384. 15741574 10.1093/jnci/dji057

[44] Moorthy B, Sriram P, Randerath E, Randerath K (1997) Effects of cytochrome P450 inducers on tamoxifen genotoxicity in female mice in vivo. Biochem Pharmacol 53: 663–669. 9113085 10.1016/s0006-2952(96)00875-1

[45] Shibutani S, Ravindernath A, Suzuki N, Terashima I, Sugarman SM, et al (2000) Identification of tamoxifen-DNA adducts in the endometrium of women treated with tamoxifen. Carcinogenesis 21: 1461–1467. 10910945

[46] Lee H, Jiang F, Wang Q, Nicosia SV, Yang J, et al (2000) MEKK1 activation of human estrogen receptor alpha and stimulation of the agonistic activity of 4-hydroxytamoxifen in endometrial and ovarian cancer cells. Mol Endocrinol 14: 1882–1896. 11075819 10.1210/mend.14.11.0554

[47] Vivacqua A, Bonofiglio D, Recchia AG, Musti AM, Picard D, et al (2006) The G protein-coupled receptor GPR30 mediates the proliferative effects induced by 17beta-estradiol and hydroxytamoxifen in endometrial cancer cells. Mol Endocrinol 20: 631–646. 16239258 10.1210/me.2005-0280

[48] Konecny GE, Pegram MD, Venkatesan N, Finn R, Yang G, et al (2006) Activity of the dual kinase inhibitor lapatinib (GW572016) against HER-2-overexpressing and trastuzumab-treated breast cancer cells. Cancer Res 66: 1630–1639. 16452222 10.1158/0008-5472.CAN-05-1182

[49] Chu I, Blackwell K, Chen S, Slingerland J (2005) The dual ErbB1/ErbB2 inhibitor, lapatinib (GW572016), cooperates with tamoxifen to inhibit both cell proliferation- and estrogen-dependent gene expression in antiestrogen-resistant breast cancer. Cancer Res 65: 18–25. 15665275

[50] Amant F, Moerman P, Neven P, Timmerman D, Van Limbergen E, et al (2005) Endometrial cancer. Lancet 366: 491–505. 16084259 10.1016/S0140-6736(05)67063-8

[51] Acconcia F, Barnes CJ, Kumar R (2006) Estrogen and tamoxifen induce cytoskeletal remodeling and migration in endometrial cancer cells. Endocrinology 147: 1203–1212. 16339197 10.1210/en.2005-1293

