# Supplementary figures and images for: Estradiol and Tamoxifen Induce Cell Migration through GPR30 and Activation of Focal Adhesion Kinase (FAK) in Endometrial Cancers with Low or without Nuclear Estrogen Receptor α (ERα)
Source: PLoS One. 2013 Sep 9;8(9):e72999. doi: 10.1371/journal.pone.0072999 (PMC3767783; doi:10.1371/journal.pone.0072999)

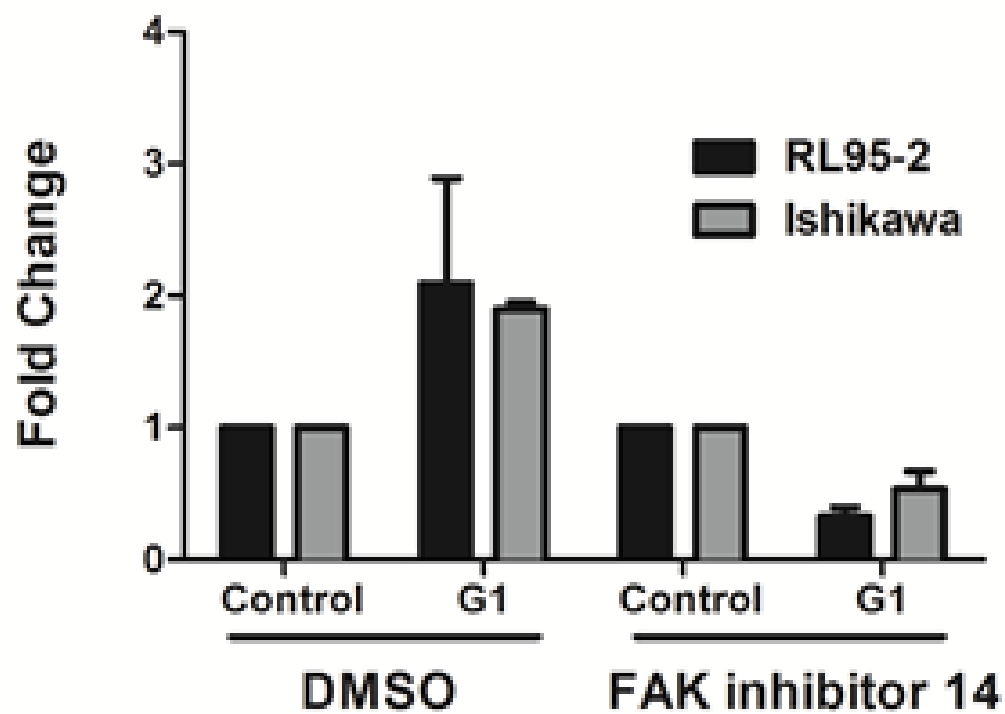

Supplement: Figure S1 — Expression of c-fos mRNA induced by G1 (a GPR30 agonist) in endometrial cancer cell lines (RL95-2 and Ishikawa) in the absence or presence of FAK inhibitor 14. To evaluate whether the activation of FAK play a role in the regulation of GPR30 target genes, endometrial cancer cells were pre-treated with FAK inhibitor 14 (15 μM) for 2 hr followed by administration of 1 µM G1 for 1 hr. Then, mRNA of c-fos in endometrial cancer cells was determined by Taqman real-time quantitative polymerase chain reaction (PCR). Expression of c-fos mRNA induced by G1 was abolished in the presence of FAK inhibitor 14 in both RL95-2 and Ishikawa endometrial cancer cells. Results shown were obtained from three independent experiments and are presented as mean + standard deviation (SD). (PDF) [file pone.0072999.s001.pdf]

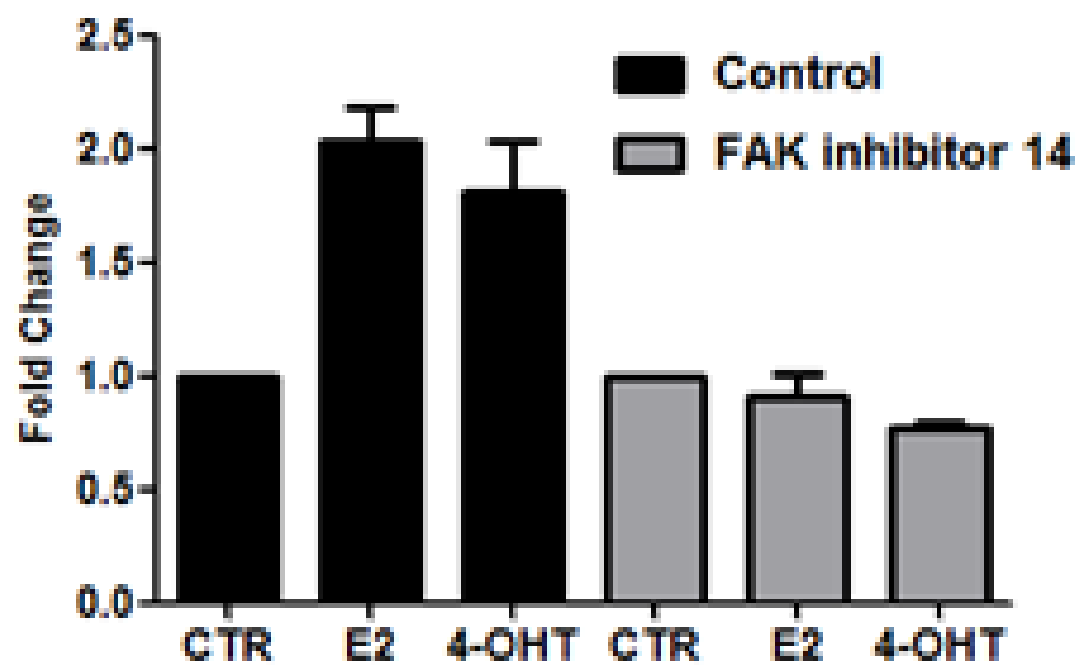

Supplement: Figure S2 — FAK inhibitor 14 blocked cell proliferation induced by 17β-estradiol (E2) and 4-hydroxytamoxifen (OHT) in ERα-negative endometrial cell RL95-2. BrdU incorportation assay was used as an index for cell proliferation. RL95-2 cells were treated with E2 or OHT (1 μM) in presence or absence of FAK inhibitor 14 (1 μM) for 48 hr. Consequently, cell proliferation of RL95-2 induced by E2 or OHT was substantially repressed in presence of FAK inhibitor 14. Results shown were obtained from three independent experiments and are presented as mean + standard deviation (SD). (PDF) [file pone.0072999.s002.pdf]
